# Supplementary material for: Splice-Junction-Based Mapping of Alternative Isoforms in the Human Proteome
Source: Cell Rep. Author manuscript; Available in PMC 2020 Jan 15. (PMC6961840; doi:10.1016/j.celrep.2019.11.026)
Supplement: 2 [file NIHMS1546469-supplement-2.zip › DF1/PXD006675/AtrialSeptum/AtrialSeptum_16_TNS1_PHNPADILLHPTGEEDEGK.pdf]

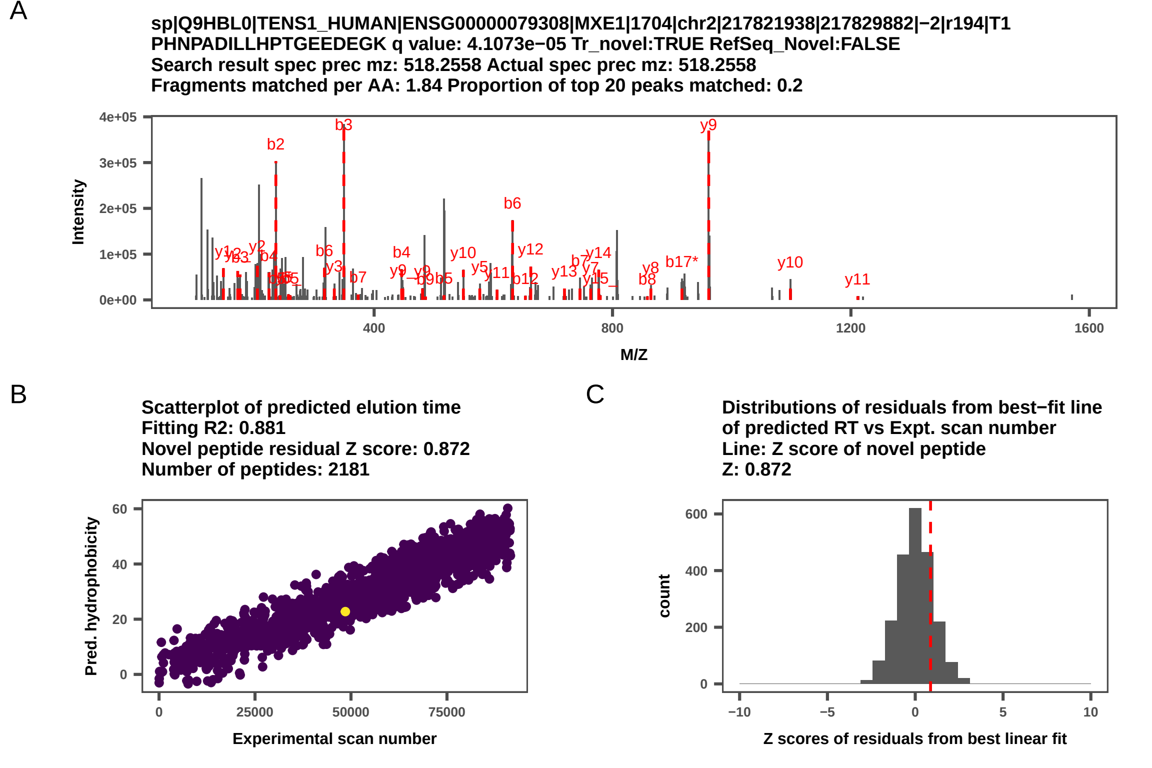
A
sp|Q9HBL0|TENS1_HUMAN|ENSG00000079308|MXE1|1704|chr2|217821938|217829882|−2|r194|T1
PHNPADILLHPTGEEDEGK q value: 4.1073e−05 Tr_novel:TRUE RefSeq_Novel:FALSE
Search result spec prec mz: 518.2558 Actual spec prec mz: 518.2558
Fragments matched per AA: 1.84 Proportion of top 20 peaks matched: 0.2
Intensity
4e+05
3e+05
2e+05
1e+05
0e+00
M/Z
400
800
1200
1600
b2
b3
y9
b6
b4
b7
y10
y12
y14
b17*
y10
y11
y13
y8
b8
B
Scatterplot of predicted elution time
Fitting R2: 0.881
Novel peptide residual Z score: 0.872
Number of peptides: 2181
Pred. hydrophobicity
60
40
20
0
Experimental scan number
0
25000
50000
75000
C
Distributions of residuals from best−fit line
of predicted RT vs Expt. scan number
Line: Z score of novel peptide
Z: 0.872
count
600
400
200
0
−10
−5
0
5
10
Z scores of residuals from best linear fit
